# Sparse frame selection for graph-based deadlift form assessment

**DOI:** 10.1038/s41598-026-67912-0

**Published:** 2026-09-11

**Authors:** Omar Shoaib, Nada A. Attia, Zitong Yu, Ghada Khoriba, Tamer Arafa

**Affiliations:** 1 Center for Informatics Science (CIS), School of IT and Computer Science, Nile University, Sheikh Zayed City, Giza Egypt; 2 Faculty of Computers and Artificial Intelligence, Helwan University, Cairo, Egypt; 3 Great Bay University, Dongguan, China

**Keywords:** Deadlift form assessment, Exercise evaluation, Computer vision, Graph convolutional networks, Pose estimation, Frame selection, Real-time analysis, Fitness technology, Engineering, Health care, Mathematics and computing

## Abstract

Automated movement analysis from monocular RGB video can enhance injury prevention and performance assessment in strength training. Yet, existing approaches struggle to accurately evaluate the deadlift—one of the most widely practiced exercises with a high risk of injury when performed incorrectly. Assessing form from monocular RGB video remains challenging due to subtle biomechanical errors, redundant frames, and the computational demands of video analysis. This study introduces a Real-Time framework that integrates the modified Search–Map–Search frame-selection method with graph-based models, trained on a custom dataset that was created, labeled as good or bad form, and annotated by a certified expert for deadlift assessment. By selecting six representative frames per repetition, the proposed frame-selection method reduces redundancy by 89.8% while preserving critical biomechanical information. Benchmark results show that the proposed Temporal Graph Convolutional Network (*proposed T-GCN*) achieved 89.5% accuracy with high precision and recall; its graph-classification stage ran in 0.4 s, while the full video-to-decision pipeline ran in approximately 0.8 s on a desktop GPU. The lightweight proposed Spatio-Temporal Graph Convolutional Network (*proposed ST-GCN*) variant, with only 4,514 parameters, achieved comparable performance (85.3% accuracy). These findings demonstrate that the proposed framework achieves competitive accuracy while substantially reducing the number of processed frames and computational cost, enabling practical deployment on commodity RGB hardware.

## Introduction

The deadlift is a popular compound, multi-joint resistance exercise that involves lifting a barbell from the ground to thigh height and lowering it back down, which effectively engages the thigh, hip, back, and core muscles^1^. Weight training, particularly deadlifting, can lead to various injuries when performed with improper technique, with 22% of competitive deadlifters (11 out of 50) attributing their injuries to poor lifting technique or posture^2^. Assessing deadlift form is challenging, as the difference between a safe and risky lift can depend on only a few degrees in hip or spine angles (e.g., slight rounding of the lower back or incorrect hip positioning), which can increase injury risk. While expert supervision is essential for enhancing performance and preventing injuries, many individuals lack access to real-time coaching to ensure correct deadlift form^3^.

Automated form assessment using video analysis could provide valuable feedback to a wider range of lifters, but accurately evaluating technique from monocular RGB video remains challenging due to subtle biomechanical deviations, viewpoint-dependent ambiguity, and the computational demands of processing full video sequences. Furthermore, repetitive motions generate redundant video frames that increase computational load without contributing meaningful information. Real-world deployment in gyms requires low-latency processing on commodity hardware, while depth sensors and wearable devices are often impractical in most training settings. These challenges highlight a gap in current solutions: an approach is needed that is both accurate for subtle form errors and efficient enough for real-time deployment using standard single-camera RGB video.

**Fig. 1:**
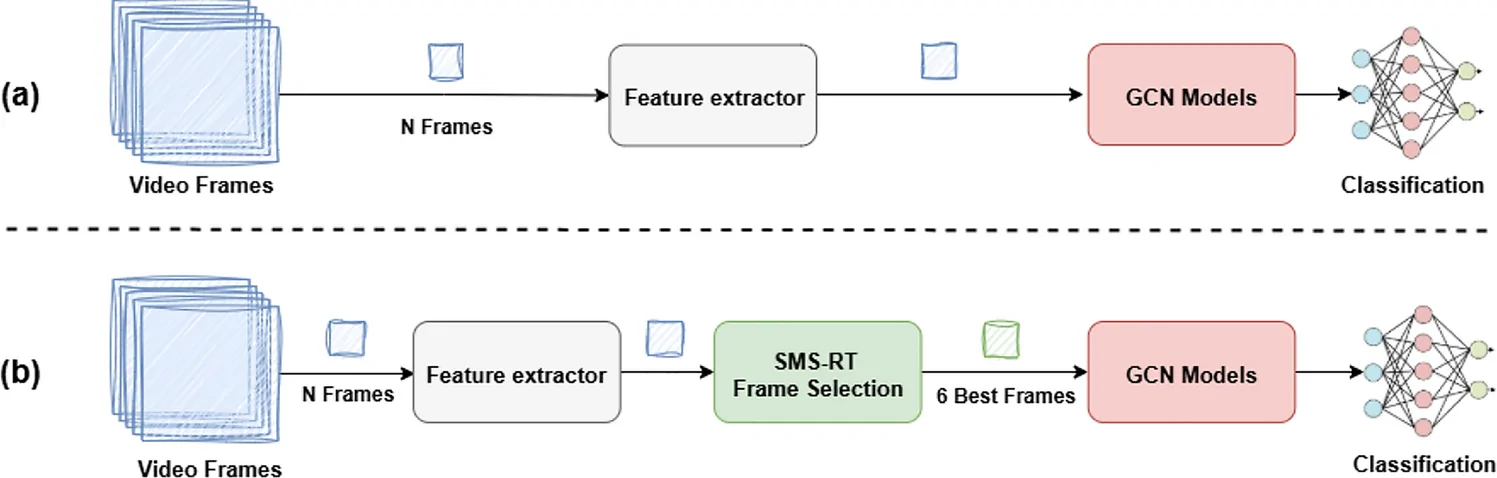
(**a**) Baseline pipeline using all frames. (**b**) Proposed pipeline with SMS-RT frame selection.

To address this gap, we propose a real-time pipeline for assessing deadlift form from monocular RGB video. Figure 1 contrasts the baseline full-sequence pipeline with our SMS-RT–based pipeline. Our main contributions are: (1) a novel expert-annotated dataset of deadlift videos labeled for form quality; (2) an efficient frame-selection pipeline using SMS-RT that replaces time-consuming manual sampling, reducing redundancy and improving scalability; and (3) a lightweight architecture that combines YOLOv11-based pose estimation with graph convolutional network (GCN) models trained on sparse, information-rich frames. The proposed system captures subtle hip–spine alignment errors critical to deadlift safety and improves inference efficiency at competitive accuracy, supporting practical deployment on commodity RGB hardware.

## Related work

### Sparse video representation

The field of automated exercise assessment using computer vision has evolved through several key technological advances. Early approaches to human action recognition employed hand-crafted features and traditional machine learning methods^4,5^. However, the introduction of deep learning fundamentally transformed this landscape, enabling more sophisticated analysis of human movement patterns^6–8^. Building on these advances, contemporary research has increasingly focused on specialized applications that address the unique challenges of exercise form assessment.

Deep learning approaches to human action recognition have progressed from basic classification to more exercise-specific form assessment^9–11^. Modern systems leverage convolutional neural networks^10,12,13^ and recurrent architectures^6,14^ to capture temporal dynamics in movement, while multimodal approaches combining physiological and visual signals have shown promise for richer fitness monitoring. Specialized datasets such as Fitness-AQA enabled more targeted workout assessment, but they focus on a limited set of exercises and primarily emphasize exercise-oriented representation learning under challenging gym conditions rather than sparse inference for real-time deployment. More recent datasets, such as EgoExo-Fitness and FLEX, further broaden the problem through egocentric/exocentric, multiview, and multimodal annotations for fitness understanding and action quality assessment. However, these systems are oriented toward broader benchmarking and richer supervision, often at the cost of increased sensing or modeling complexity. A recent review also noted that pose-based movement-feedback pipelines still vary substantially in how movement quality is modeled and communicated, making method selection for new exercise settings non-trivial. These limitations are especially relevant for deadlift assessment, where the goal is not broad activity understanding, but accurate screening of subtle form faults under strict latency constraints^9,15–19^.

The computational burden of processing entire video sequences has consequently driven the development of intelligent frame selection methods^20–22^. Uniform and random sampling represent baseline approaches, while advanced methods employ motion-based filtering^21,23,24^ and hierarchical selection strategies^20,25^. Recent adaptive sampling techniques utilize motion-driven selection^26,27^ and redundancy-aware clustering^22,24^ to identify information-dense frame subsets while maintaining temporal coherence. These frame selection innovations create the foundation for efficient video processing, but their value in exercise assessment depends on whether sparse subsets can still preserve the biomechanically discriminative moments required for reliable form classification.

### Skeleton-based action recognition

Building upon efficient frame selection, human pose estimation methods divide into top-down and bottom-up approaches^28–30^. Multi-stage methods like OpenPose provide exceptional accuracy through sophisticated processing pipelines^28,31,32^ but require significant computational resources. Single-stage detectors including YOLO-based variants^29,33,34^ achieve real-time performance suitable for interactive applications while maintaining reasonable accuracy. Recent advances focus on balancing precision with computational efficiency for practical deployment scenarios^30,35^. However, pose-based exercise systems remain sensitive to viewpoint variation, occlusion, clothing, and background clutter, all of which are common in gym environments^9,19^.

Exercise-specific systems built on pose representations also differ substantially in objective from the present work. Fitness-AQA emphasizes expert-labeled workout error detection and representation learning in real gym scenarios, LiFT targets large-scale exercise detection and repetition counting from 3D human-pose representations, and BioCoach focuses on biomechanics-grounded, language-based coaching from streaming video. Recent deadlift-focused work has also explored classifying deadlift styles such as conventional, Romanian, and sumo from video input. In contrast, our method focuses specifically on expert-labeled good-versus-bad deadlift form assessment from monocular RGB video, explicit reduction of temporal redundancy to six representative frames, and graph-based modeling of sparse pose sequences for efficient real-time deployment^9,36–38^.

Addressing the need for structured joint relationship modeling, Graph Convolutional Networks have revolutionized skeleton-based action recognition by representing the human body as a graph of connected joints^39–41^. Foundational methods like ST-GCN established spatial-temporal convolution frameworks^39^, while adaptive approaches enabled learnable graph topologies^40,42,43^. Advanced architectures incorporate attention mechanisms^43,44^, multi-stream processing^40,45^, and specialized modules for enhanced feature learning^41,42^. These graph-based methods effectively capture both spatial relationships between joints and temporal motion dynamics, making them well suited to exercise assessment where subtle multi-joint coordination errors determine movement quality.

The convergence of efficient frame selection, pose estimation, and graph-based modeling creates the foundation for automated exercise assessment systems. In the specific case of deadlift analysis, this integration is especially important because practical systems must preserve subtle biomechanical cues while remaining efficient enough for real-time use on standard RGB hardware.

## Methods

**Fig. 2:**
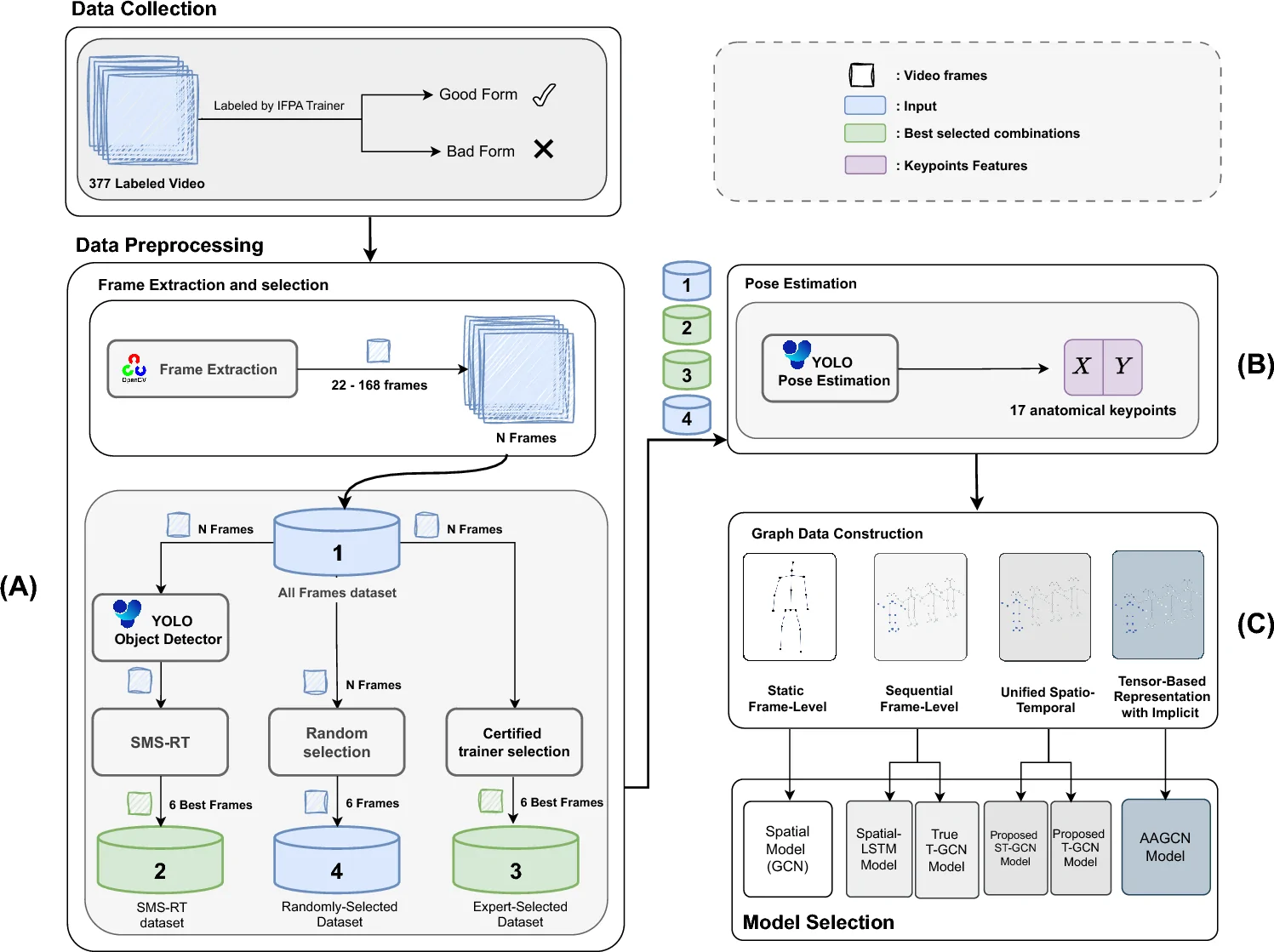
Workflow pipeline illustrating the steps from expert-labeled deadlift video collection to SMS-RT frame selection, pose estimation, graph construction, and GCN-based classification.

### Data collection

In this study a custom dataset of 377 video clips was created under expert supervision specifically for deadlift form assessment addressing the lack of annotated data for strength training exercises. Each clip captures a single deadlift repetition performed by one of three male participants (aged 19–25) with intermediate training experience. Participants performed deadlifts with a wide range of weights from an empty PVC pipe to loaded barbells up to 200 kg allowing the dataset to cover varied effort levels. The video recordings were taken using two smartphones from five different angles covering both frontal and diagonal views of the movements. Each internal video clip was labeled by a certified International Fitness Professionals Association (IFPA) trainer as either good or bad form using predefined biomechanical criteria, including joint alignment, hip movement, spinal posture, and bar control. Each repetition was assessed independently and blinded to the model outputs and study hypotheses.

To probe robustness beyond the internal cohort, an evaluation-only external set was curated from publicly available Kaggle fitness video datasets^46,47^. From these sources, 54 deadlift repetitions were selected under diverse viewpoints, lighting, and background conditions, drawn from 16 lifters (14 male and 2 female) with varied morphology. These external clips were reviewed using a three-annotator consensus protocol, including the original internal IFPA trainer, and retained only when consensus good-form labeling was achieved. The external samples were not used for training; instead, they were merged with the held-out internal test split to form an expanded evaluation set (N=130) used only for robustness analysis under increased real-world variability.

**Labeling rubric**. To make the good/bad criteria explicit, the certified trainer applied a conservative ”error window” protocol keyed to the deadlift phases: a repetition is labeled “bad” if a fault appears in any phase. The per-phase criteria are summarized in Table 1.

**Camera setup**. The internal recordings were captured with two consumer smartphones (an iPhone 13 and a Samsung Galaxy S9+) across five viewpoints, including one sagittal/side view and four frontal or diagonal views. Four views were recorded indoors and one outdoors to introduce lighting and background variability. The five-view setup was used to introduce viewpoint, lighting, and background variability rather than to prescribe a multi-camera deployment protocol. The specific device assigned to each view and the exact camera height and lifter-to-camera distance were not metrically standardized during collection, which is acknowledged as a limitation.

**Table 1:** Per-phase labeling rubric used by the certified IFPA trainer for the internal dataset. A repetition is labeled “bad” if any phase exhibits its listed fault.

#	Phase (bar position)	“Good” criterion	Fault *→ * “bad”
1	Bar on ground (setup/liftoff)	neutral lumbar; hips set; bar over mid-foot	rounded lower back at setup; hips too high/low
2	Initial pull below knees (first pull)	hips and shoulders rise together; neutral spine	“stripper pull” (hips shoot up, bar drifts); lumbar flexion
3	Bar at knee level (transition)	neutral spine maintained at maximum mechanical disadvantage	catastrophic lumbar flexion; bar drifts forward
4	Bar above knees (second pull)	hip drive engaged; bar close to body	knees re-bend; bar swings away
5	Mid-femur	continued hip extension; neutral spine	hyperextension begins; back rounding
6	Final standing (lockout)	full hip/knee extension; neutral spine	excessive backward lean / hyperextension at lockout

### Data preprocessing

A structured preprocessing pipeline was implemented to prepare the raw video data for model training. The pipeline consists of three sequential stages: frame extraction and selection, pose estimation and keypoint extraction, and graph data construction for spatial-temporal model training.

**Fig. 3:**
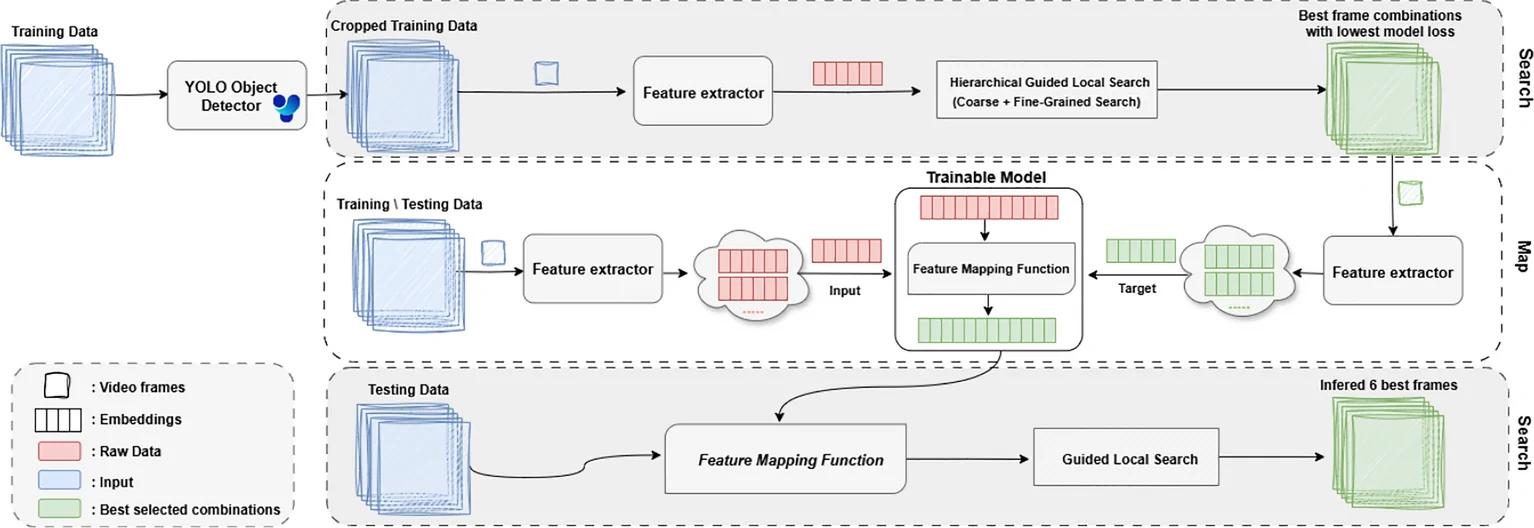
Workflow of the SMS-RT method for frame selection. The process begins with YOLOv11-based object detection and feature extraction using a ResNet50+TSN backbone. In the first “Search” stage, a hierarchical guided search identifies frame subsets with minimal loss. The “Map” stage employs a Transformer-based function to learn the mapping between video features and optimal frame sets. The final “Search” stage applies this function to unseen data to infer six informative frames.

#### Frame extraction and selection

**Fig. 4:**
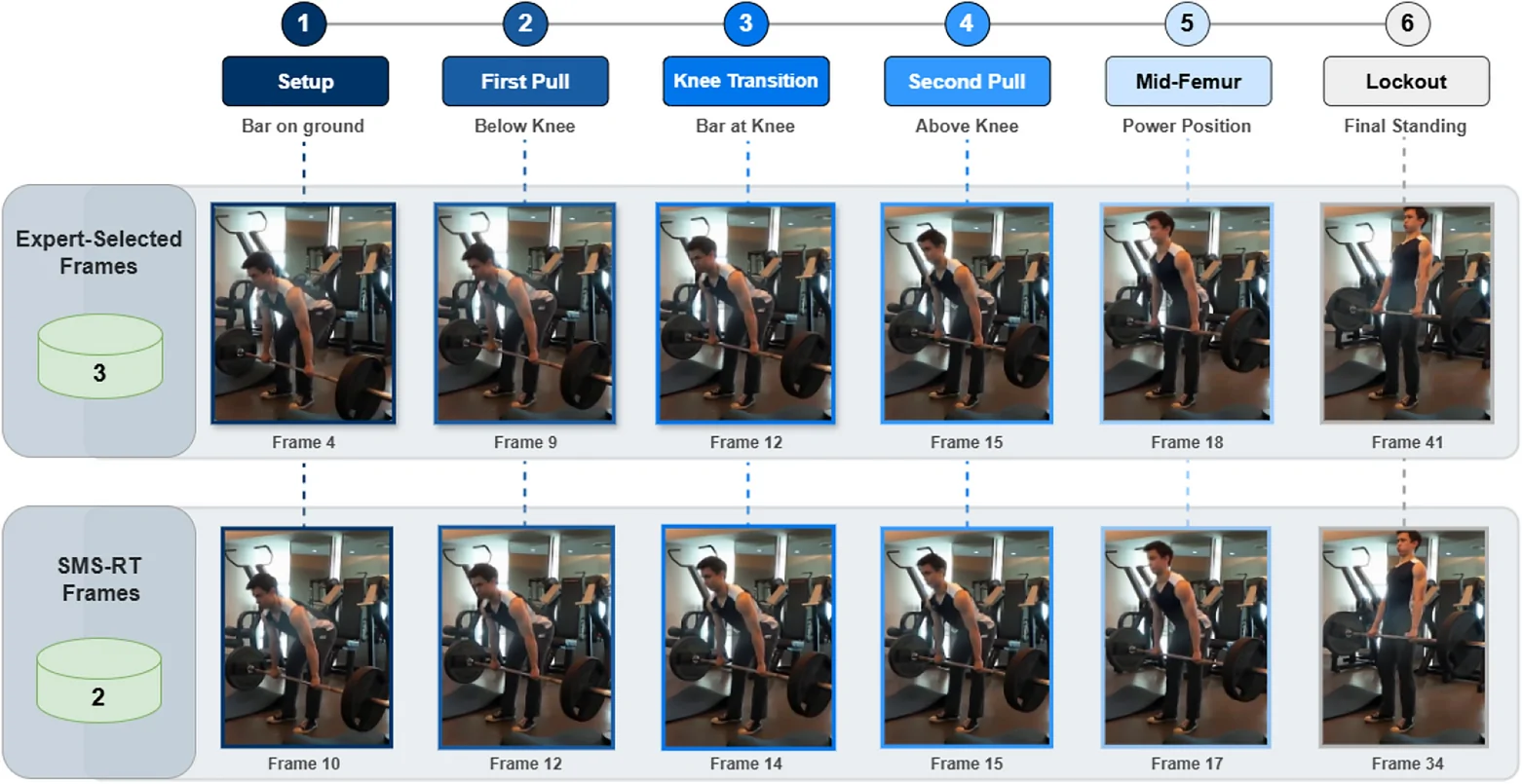
SMS-RT-selected frames align closely with expert-defined deadlift phase checkpoints (setup to lockout), providing qualitative support for biomechanical phase-based selection.

The collected video clips were manually cropped to isolate only the complete deadlift movements. Individual frames were then extracted from each video using OpenCV^48^ and assigned unique identifiers (e.g., clip1_frame1, clip1_frame2, etc.) to form the *All Frames Dataset*, which preserves the full temporal sequence with variable frame counts per video (22–168 frames, depending on duration and frame rate). To reduce redundancy while maintaining classification accuracy, three additional datasets were derived from the All Frames Dataset using distinct frame-selection strategies as shown in Fig. 2A. The first, termed the *SMS-RT Dataset*, applies a modified Search–Map–Search method^20^ to extract exactly six representative frames per video through a three-stage process illustrated in Fig. 3. In the first stage, Feature Extraction, YOLOv11^34^ detects bounding boxes, followed by a ResNet-50 backbone^49^ combined with Temporal Segment Networks (TSN)^50^ to extract 2048-dimensional features. The second stage, Hierarchical Search, performs coarse-grain sampling to gather a diverse pool of frames and then applies fine-grain optimization to select the six frames that minimize classification loss. The final stage, Feature Mapping, employs a Transformer-based module^51^ to learn the mapping between video-level features and the optimal six-frame representation, enabling efficient inference. The second dataset, the *Randomly Selected Dataset*, uniformly samples six frames at random from each video, serving as an uninformed baseline. The third dataset, the *Expert-Selected Dataset*, is manually curated by a certified IFPA trainer who selects six frames per video based on key biomechanical checkpoints corresponding to the bar’s position: (1) bar on ground, (2) initial pull below knees, (3) bar at knee level, (4) bar above knees, (5) mid-femur position, and (6) final standing position. The six selected frames correspond to phase-oriented checkpoints of the deadlift, including biomechanically important instants such as liftoff, knee passage, and lift completion, where joint configuration, bar position, and trunk–hip coordination change most distinctly (Fig. 4)^52–55^.

#### Pose estimation and keypoint extraction

Following the frame selection process, each frame from the four datasets was processed using the YOLOv11-Pose model^34^ for 2D human pose estimation as shown in Fig. 2B. For each frame, the model detects the primary subject and extracts 17 anatomical keypoints corresponding to the Common Objects in Context (COCO) standard^56^, which includes major joints such as the shoulders, elbows, hips, knees, and ankles.

The raw pixel coordinates for each of the 17 keypoints are then normalized by the full video frame’s width and height, respectively, to produce scale-invariant (*x*, *y*) coordinates. This representation captures projected 2D kinematic cues only; therefore, it cannot directly recover true depth-sensitive biomechanical quantities such as 3D bar path or spinal curvature. The learning signal is derived from normalized 2D pose keypoints (17-joint graphs) rather than raw RGB frames, reducing appearance bias and emphasizing kinematic structure. These 17 two-dimensional coordinate vectors for each frame are then organized and stored in CSV format. This structured dataset, capturing only the essential spatial configuration of the body, serves as the direct and sole input for the subsequent graph construction stage.

#### Graph data construction

Following keypoint extraction, the raw temporal sequence of 17 2D coordinates for each video is transformed into a graph-based representation suitable for model ingestion. A foundational element for all methods is the set of 17 keypoints serving as graph nodes, where each node’s feature vector consists of its normalized (x, y) coordinates. The connections between these nodes are defined differently depending on the model’s architecture, resulting in four distinct data construction paradigms as shown in Fig. 2C.

**Static Frame-Level Graphs**. For the baseline *Spatial GCN* model, which lacks a temporal component, every frame across all videos is treated as an independent data point. Each frame is converted into a discrete graph containing 17 nodes and a fixed set of 32 undirected edges representing the human skeletal structure, following established pose connectivity conventions^28,39^. The label (“good” or “bad” form) of the source video is assigned to each of its constituent frame-graphs. This method isolates the purely spatial characteristics of a single pose.

**Sequential Frame-Level Graphs**. To incorporate temporal dynamics for the *Spatial LSTM*^43,57^ and *T-GCN* models, videos are represented as ordered sequences of the static frame-level graphs described above. Each item in the dataset corresponds to one video, structured as a sequence of graphs. This format preserves the temporal evolution of poses, allowing the models’ recurrent layers (LSTM or GRU) to learn patterns from the sequence of spatial features. The graph structure itself does not contain explicit temporal edges; temporality is modeled by the architecture^43,57^.

**Unified Spatio-Temporal Graphs**. For the *Proposed ST-GCN* and *Proposed T-GCN* models, each video is converted into a single, large-scale spatio-temporal graph that explicitly encodes both spatial and temporal relationships^39,40^. For a video with *T* frames, this “giant graph” contains *17 × T* nodes, where each node represents a specific joint at a specific frame. The graph’s connectivity is defined by two distinct edge types: (1) *spatial edges*, which apply the standard skeletal connections within each of the *T* frames, and (2) *temporal edges*, which connect instances of the same joint across consecutive frames (e.g., linking the left knee at frame *t* to the left knee at frame *t+1*). This construction allows the models to learn complex spatio-temporal patterns via message passing across both edge types^39,40^.

**Tensor-Based Representation with Implicit Graphs**. The *AAGCN* model employs a different paradigm that does not rely on explicit graph objects. Instead, each video’s keypoint data is formatted into a dense tensor of shape *[C, T, V]*, where *C=2* is the number of coordinate channels, *T* is the number of frames, and *V=17* is the number of joints. To handle variable-length videos, sequences are padded to a uniform length *T*. In this setup, the graph structure is implicitly defined within the model itself. The AAGCN layer dynamically learns an adaptive adjacency matrix from the input data, capturing both spatial and temporal relationships through attention mechanisms^40,42^. For reproducibility, graph construction is deterministic once the split seed and selected frames are fixed: coordinates are normalized by frame width and height, spatial edges follow a fixed 17-joint COCO skeleton, temporal edges connect identical joints across adjacent frames, and tensor-based inputs are padded using a consistent rule for sequence alignment.

### Model architectures

Six graph-based models were developed to classify deadlift form from pose data. Each model leverages spatial or spatio-temporal information differently, drawing from state-of-the-art designs in skeleton-based action recognition.

**Spatial Graph Convolutional Network Model** was designed to classify individual frames based on the spatial configuration of body joints. Each input graph consisted of 17 nodes corresponding to anatomical keypoints, where each node was represented by a 2D feature vector containing normalized *x* and *y* coordinates. Fixed skeletal edges define the connections between joints, as in OpenPose and ST-GCN^28,39^. The model focuses purely on spatial pose information from a single frame without any temporal context.

**Spatial + Long Short-Term Memory (LSTM) Model** extends the Spatial GCN by adding temporal information through a sequential LSTM layer^43,57^. Each frame in the video’s temporal sequence is processed independently to extract frame-level spatial features. The resulting sequence of feature vectors is passed into an LSTM network, which learns temporal dependencies across frames.

**Temporal Graph Convolutional Network (T-GCN) Model** processes videos as sequences of individual graphs using a temporal graph convolutional network^40^. Each video is represented as a temporal sequence of frame-graphs, with graph convolutions at each time step and a gated recurrent unit (GRU) updating the hidden state based on temporal dependencies.

**Adaptive Attention Graph Convolutional Network (AAGCN) Model** applies adaptive graph convolutions that learn the optimal joint connectivity based on input features^40,42^. The model dynamically computes an adjacency matrix that captures both spatial and temporal relationships via attention mechanisms. This enables adaptive modeling of complex movement patterns during classification and improves generalization to varying movement forms.

**Proposed ST-GCN and T-GCN Models** represent each video as a single spatio-temporal graph, where each node corresponds to a joint at a specific frame^39,40,43^. Spatial edges connect anatomical joints within the same frame, while temporal edges link the same joint across consecutive frames. The ST-GCN variant uses ChebConv, while the T-GCN uses GCNConv, for graph convolutional operations. The learned spatio-temporal representation is aggregated and passed to a classification layer, enabling the capture of complex inter-frame dynamics required for robust form assessment.

### Experimental setup and training protocol

To ensure fair comparison and reproducibility across all evaluated architectures and frame-selection paradigms, a unified training and evaluation protocol is adopted. Data splitting is performed at the video level to prevent leakage. The dataset is partitioned into 301 training videos (80%) and 76 held-out test videos (20%). The training split contains 1,098 good-form and 708 bad-form frame-level samples, while the held-out test split contains 276 good-form and 180 bad-form frame-level samples. To assess stability and support model selection, 5-fold stratified cross-validation is conducted on the training split while keeping the test split untouched for final reporting. Standardized optimization settings are summarized in Table 2. In addition to the internal held-out test split, an expanded evaluation set is formed by merging the held-out test videos with the external Kaggle evaluation set (N=130) for robustness analysis. To further assess subject-level generalization, a subject-independent leave-one-subject-out (LOSO) evaluation was also performed. In each fold, one participant was held out for testing while the models were trained on the remaining two participants. Because one participant performed only good-form repetitions, that fold is single-class and is excluded from the accuracy comparison. This evaluation was conducted for both All-frames and SMS-RT inputs, with the SMS-RT selector retrained within each fold to avoid using held-out subject data during frame selection.

**Table 2:** Unified optimization and training configuration used across all models.

Component	Setting
Loss	Cross-entropy
Optimizer	Adam
Initial learning rate	$1\times 10^{-3}$
LR scheduler	ReduceLROnPlateau (factor 0.5, patience 7)
Max epochs	100
Early stopping	patience 15
Cross-validation	5-fold stratified CV on training split only
Random seed	42 (NumPy/PyTorch and split operations)
Hardware	NVIDIA RTX 3060 (12GB VRAM)
Batch size	32 / 16 / 8 / 1 (by model family)

**Latency measurement**. Inference latency was measured with GPU synchronization on an NVIDIA RTX 3060 desktop GPU. We report both graph-classification latency and end-to-end pipeline latency. Graph-classification latency measures only the final GCN-based prediction stage after pose keypoints have been extracted and converted to graph inputs. End-to-end latency includes video decoding, feature extraction for SMS-RT frame selection, SMS-RT selection, YOLOv11-Pose estimation on the selected frames, graph construction, and graph classification.

## Results

The following results demonstrate the effectiveness of intelligent frame selection and graph-based modeling in classifying deadlift form with high accuracy and efficiency. This section is organized as follows: Section ”SMS-RT frame selection performance” reports the SMS-RT frame-selection results; Section “Model performance on SMS-RT dataset” presents the classification performance across all models using SMS-RT-selected frames; Section “Ablation study: impact of frame selection and proposedmodels” provides an ablation study comparing SMS-RT with full-frame and fixed-frame strategies; Section “External robustness on expanded test set” evaluates robustness on the expanded external test set; Section “Subject-independent evaluation” reports subject-independent leave-one-subject-out performance; End-to-end inference latency is reported with the model-performance results in Section “Model performance on SMS-RT dataset”.

### SMS-RT frame selection performance

The SMS-RT frame selection pipeline successfully addressed the computational challenge posed by variable-length deadlift videos (22–168 frames per clip). Table 3 summarizes the performance metrics across the three training stages.

**Table 3:** SMS-RT frame selection pipeline performance (averaged across 377 training videos).

Stage	Metric	Value	Interpretation
Stage 1: Feature extraction	Video-level accuracy	44.9% ± 31.1%	High variance across videos
	Video-level loss	0.704 ± 0.050	Low variance; stable
Stage 2: Hierarchical search	Frame reduction rate	89.8%	mean per-video reduction
	Frames before (range)	22–168 (mean: 77.9)	Variable-length input
	Frames after	6 (constant)	Fixed-length output
Stage 3: Mapping function	Final training loss	0.049	95.1% similarity$^{\dagger }$
	Final validation loss	0.067	93.3% similarity$^{\dagger }$
	Generalization gap	0.018	Robust to overfitting

$^{\dagger }$Cosine similarity = 1 - loss

The ResNet-50+TSN feature extraction stage (Stage 1) achieved a mean accuracy of 44.9% ± 31.1% with a loss of 0.704 ± 0.050. While the high standard deviation reflects the inherent variability in video difficulty and recording conditions, the low loss variance (± 0.050) demonstrates stable feature extraction across the dataset, providing a reliable foundation for subsequent frame selection.

The hierarchical search algorithm (Stage 2) achieveda mean per-video frame reduction of 89.8%, reducing an average of 77.9 frames per video to six representative frames. This dramatic reduction eliminated temporal redundancy while preserving biomechanically critical moments (setup, pull initiation, knee-level transition, lockout), as evidenced by the improved downstream classification performance (Section “Ablation study: impact of frame selection and proposed models”).

The Transformer-based mapping function (Stage 3) demonstrated strong learning convergence, achieving a final training loss of 0.049 (equivalent to 95.1% cosine similarity between predicted and optimal frame representations) and a validation loss of 0.067 (93.3% similarity). The minimal generalization gap of 0.018 between training and validation losses indicates robust learning without overfitting, confirming the model’s ability to generalize its frame selection strategy to unseen videos. This learned mapping enables efficient inference on new data without requiring exhaustive search for each video.

**Fig. 5:**
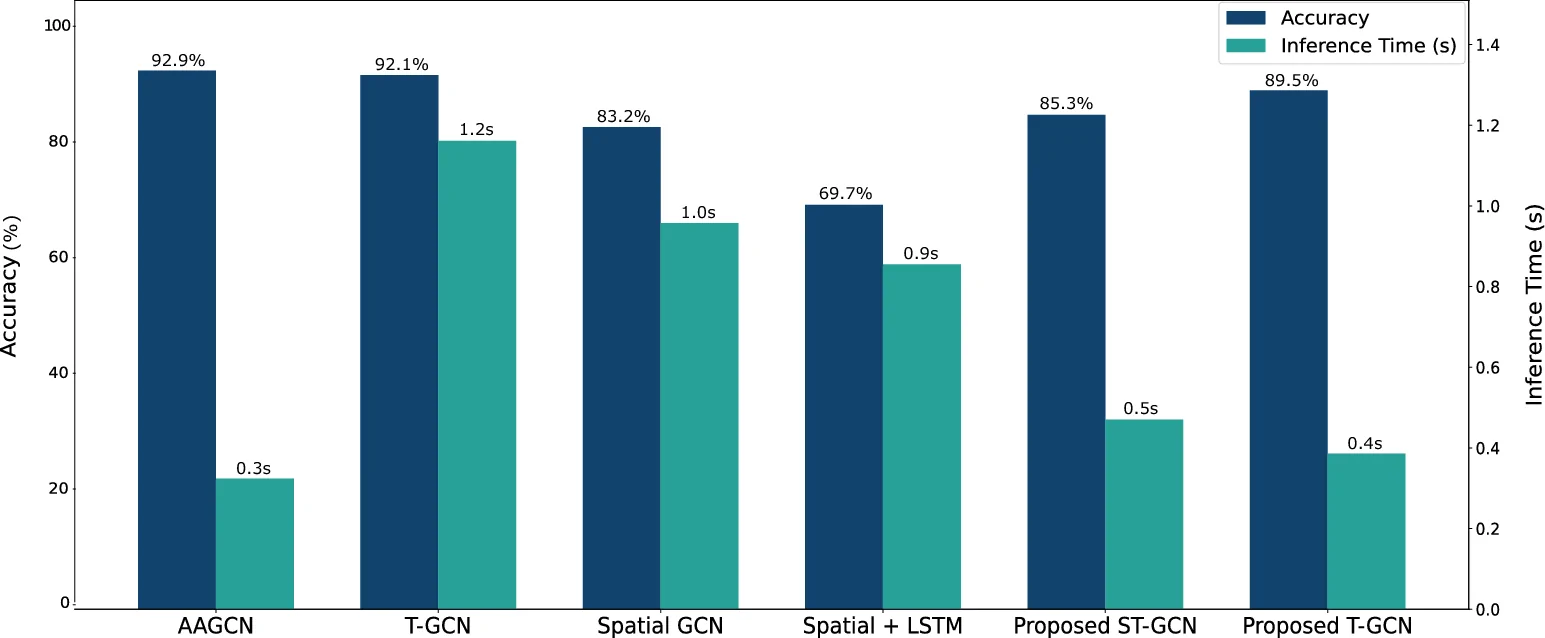
Accuracy and inference time comparison of all GCN models on the SMS-RT dataset.

### Model performance on SMS-RT dataset

The classification performance of six graph-based models was assessed using the SMS-RT dataset, which contains six informative frames per video selected via the proposed frame reduction strategy. Our comparison is intended to characterize the practical speed–accuracy trade-off for real-time deployment, rather than claim absolute architectural superiority. As shown in Table 4, Adaptive Attention Graph Convolutional Network (AAGCN) achieved the best performance with an accuracy of 92.9% and an F1-score of 93.2%. The proposed T-GCN performed competitively with 89.5% accuracy and an 89.8% F1-score followed by the proposed ST-GCN with 85.3% accuracy and 86.1% F1-score. In contrast, the Spatial + LSTM model showed the lowest performance across all metrics with an accuracy of 69.7% and an F1-score of 74.5%. In terms of efficiency, although T-GCN achieved high performance, it required the longest training of 385.0s and inference times of 1.2s. The proposed models showed a trade-off between accuracy and computational cost. The proposed T-GCN had the shortest training time of 8.3s and inference time of 0.4s. Meanwhile, the proposed ST-GCN was the most lightweight model with only 4,514 parameters making it ideal for deployment. Figure 5 visualizes the trade-off between accuracy and inference time across models highlighting the effectiveness of the proposed models for real-time applications.

Beyond the per-model classification times, the full inference pipeline was profiled end to end with GPU synchronization on a commodity desktop GPU (Table 5). The full pipeline ran in approximately 0.8 s per repetition. The dominant cost was the feature-extraction pass over all frames required for frame selection, followed by video decoding and pose estimation on the six selected frames; the graph-based classification forward pass itself ran in under 3 ms. Therefore, the 0.4-0.5 s figures reported for the proposed models in Table 4 are per-repetition model inference times measured in the evaluation harness, distinct from both the isolated classification forward pass and the full video-to-decision pipeline.

**Table 4:** Performance metrics for six graph-based models using SMS-RT dataset.

Model	Accuracy *↑ * (%)	Precision *↑ * (%)	Recall *↑ * (%)	F1-score *↑ * (%)	Params *↓ *	TT (s) *↓ *	IT (s) *↓ *
AAGCN	***92.9***	89.9	***96.8***	***93.2***	45,544	20.8	***0.3***
T-GCN	92.1	***90.2***	95.3	92.4	25,474	385.0	1.2
Spatial GCN	83.2	76.8	95.5	85.1	6,690	17.5	1.0
Spatial + LSTM	69.7	67.1	85.8	74.5	25,810	158.6	0.9
**Proposed ST-GCN (ours)**	85.3	81.4	91.6	86.1	***4,514***	10.5	0.5
**Proposed T-GCN (ours)**	89.5	87.3	92.6	89.8	58,818	***8.3***	0.4

TT: Training time, IT: Inference time. Best-performing values are shown in bold italics.

**Table 5:** End-to-end inference latency per repetition on a commodity desktop GPU (NVIDIA RTX 3060), measured with GPU synchronization.

Stage	Time (ms)
Feature extraction (all frames)	447
Video decoding	201
Pose estimation (6 selected frames)	99
Frame selection (SMS-RT)	88
Graph classification (GCN)	*<3*
End-to-end	*≈ 835*

### Ablation study: impact of frame selection and proposed models

The ablation study investigates the impact of the SMS-RT frame selection strategy and assesses the consistency of the proposed models under different input conditions. It is organized into two parts. Part one compares model performance using all frames against SMS-RT-selected frames to evaluate the trade-off between accuracy and computational efficiency. Part two benchmarks SMS-RT against two fixed-frame strategies which are expert-based and random sampling to assess the reliability of its automated selection process.

**Fig. 6:**
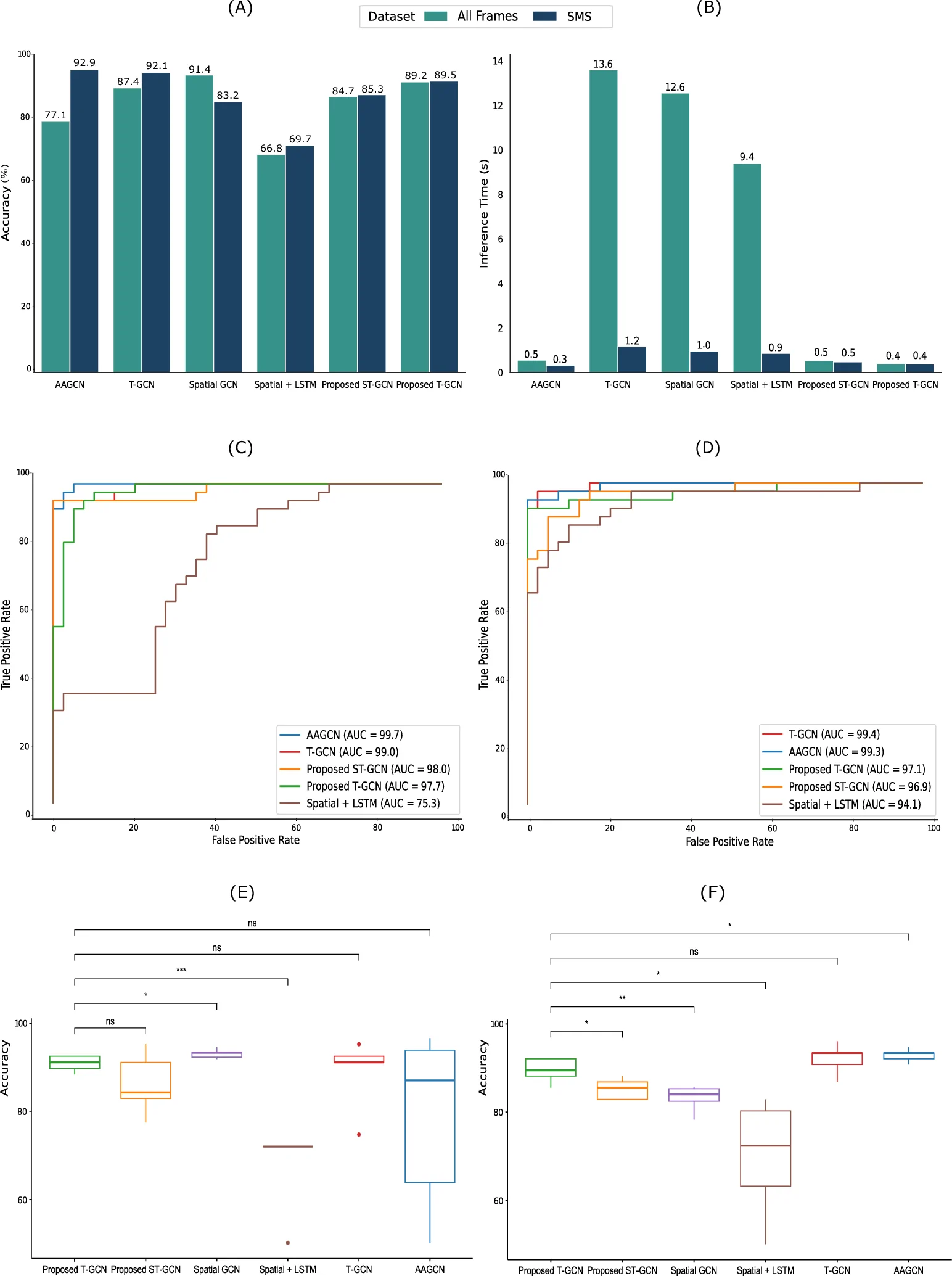
Ablation study comparing the All Frames and SMS-RT. (**A**) Accuracy comparison across models. (**B**) Inference time comparison. (**C**–**D**) ROC curves for All Frames and SMS-RT. (E–F) Statistical significance of performance differences based on cross-validation results for All Frames and SMS-RT.

#### A comparison between SMS-RT and all frames input

The six models were tested using all video frames and selecting only six informative frames with SMS-RT to understand the trade-offs between the accuracy and computational efficiency. The SMS-RT approach not only reduced the number of input frames, but also improved accuracy across all models. As shown in Fig. 6A, AAGCN’s accuracy increased from 77.1% with all frames to 92.9% using SMS-RT. T-GCN also improved from 87.4 to 92.1%. Similarly, the F1-score of AAGCN increased from 81.1 to 93.2% using fewer frames. The proposed ST-GCN and proposed T-GCN perform consistently well under both conditions. The SMS-RT approach significantly enhanced computational efficiency as T-GCN’s training time decreased from 3004.0 to 385.0 s and inference time from 13.6 to 1.2 s, while Spatial + LSTM improved from 1666.4 to 158.6 s and 9.4 to 0.9 s as in Fig. 6B. Figure 6C, D show the receiver operating characteristic (ROC) curves for the All Frames and SMS-RT, respectively. The area under the curve (AUC) values for the proposed ST-GCN and proposed T-GCN using all frames were 98.0% and 97.7%, respectively, while with SMS-RT they were still competitive at 96.9% and 97.1%. Figure 6E, F illustrate the distribution of model accuracy across five cross-validation folds for both the All Frames and SMS-RT. The SMS-RT approach resulted in not only higher accuracy but also reduced variability across folds particularly for AAGCN and T-GCN. AAGCN’s accuracy ranged from 85.5 to 94.7% with SMS-RT compared to a wider spread of 50.0% to 94.7% using all frames. T-GCN also ranged from 86.8 to 96.0% using SMS-RT and 73.7% to 93.4% with all frames. These results demonstrate that selecting a few informative frames improves both the performance and consistency of the models.

**Fig. 7:**
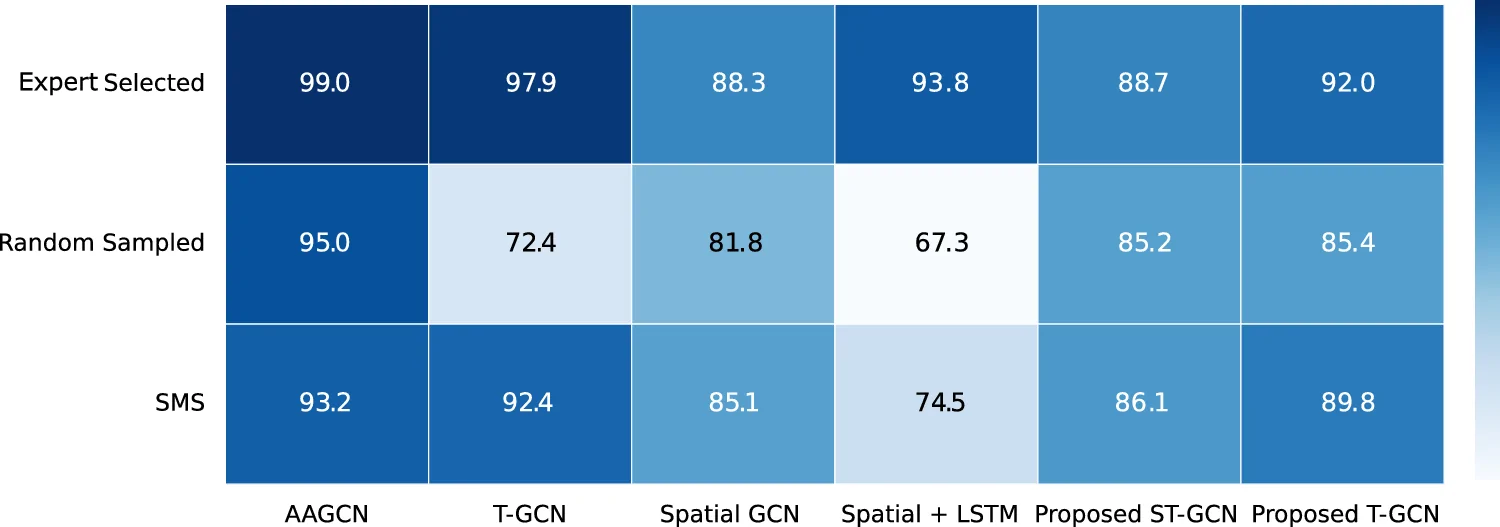
Heatmap comparison of F1-scores across models using SMS-RT, expert-selected, and randomly sampled frames.

#### A comparison between SMS-RT and fixed input strategies

To evaluate the quality and reliability of the SMS-RT-selected frames by comparing them with two fixed input strategies which are expert-selected frames and randomly sampled frames. As shown in Table 6, expert-selected frames achieved the highest overall performance across most of the models since it relies on expert knowledge to ensure clinically relevant and informative frames. AAGCN reached an accuracy of 99.0% and an F1-score of 99.0%, while T-GCN achieved 97.9% in both metrics. However, this manual method is time-consuming which takes hours or even days to annotate making it impractical for large-scale use. In contrast, SMS-RT offers a highly efficient and scalable alternative achieving F1-scores close to those of the expert selected frames as shown in Fig. 7. For instance, Proposed ST-GCN reached an F1-score of 86.1% with SMS-RT and 88.7% using expert-selected frames. Random sampling showed much lower and inconsistent performance across models. For example, T-GCN dropped to an F1-score of 72.4% and accuracy of 63.9%. The results confirm that SMS-RT is not selecting frames randomly, but rather identifying frames that enhance classification accuracy.

**Table 6:** Ablation study across different views and models.

Model	View	Accuracy *↑ * (%)	Precision *↑ * (%)	Recall *↑ * (%)	F1-score *↑ * (%)	TT (s) *↓ *	IT (s) *↓ *
AAGCN	All frames	77.1	76.6	89.5	81.1	23.2	0.5
	Expert selected	***99.0***	***98.5***	***99.5***	***99.0***	22.2	0.3
	Random sampled	94.7	93.4	96.8	95.0	25.2	***0.3***
	**SMS (ours)**	92.9	89.9	96.8	93.2	***20.8***	0.3
T-GCN	All frames	87.4	85.2	90.5	87.7	3004.0	13.6
	Expert selected	***97.9***	***97.0***	***98.9***	***97.9***	352.9	1.1
	Random sampled	63.9	61.7	91.1	72.4	***243.1***	***1.1***
	**SMS (ours)**	92.1	90.2	95.3	92.4	385.0	1.2
Spatial GCN	All frames	***91.4***	***87.0***	96.1	***91.3***	168.1	12.6
	Expert selected	86.9	81.4	***96.9***	88.3	16.8	1.1
	Random sampled	78.8	73.2	93.5	81.8	***16.2***	1.1
	**SMS (ours)**	83.2	76.8	95.5	85.1	17.5	***1.0***
Spatial + LSTM	All frames	66.8	63.3	87.9	72.9	1666.4	9.4
	Expert selected	***93.4***	***90.1***	***97.9***	***93.8***	263.3	0.8
	Random sampled	52.4	51.5	***97.9***	67.3	***127.8***	***0.8***
	**SMS (ours)**	69.7	67.1	85.8	74.5	158.6	0.9
**Proposed ST-GCN (ours)**	All frames	84.7	80.0	94.7	86.4	9.4	0.5
	Expert selected	***86.8***	***83.0***	***96.3***	***88.7***	10.9	0.5
	Random sampled	83.9	79.2	92.1	85.2	***9.2***	0.5
	**SMS (ours)**	85.3	81.4	91.6	86.1	10.5	***0.5***
**Proposed T-GCN (ours)**	All frames	89.2	87.4	92.1	89.6	14.1	0.4
	Expert selected	88.7	***88.9***	***98.4***	***92.0***	9.1	0.4
	Random sampled	83.9	78.4	93.7	85.4	9.0	***0.4***
	**SMS (ours)**	***89.5***	87.3	92.6	89.8	***8.3***	0.4

TT: Training time, IT: Inference time. Best-performing values are shown in bold italics.

### External robustness on expanded test set

Table 7 reports accuracy robustness on the expanded merged test set (N=130) relative to the original held-out test split (N=76). Under the Expert Selected view, five out of six models remained statistically stable (*p*>0.05), while AAGCN showed a significant accuracy drop (*p*=0.004). Under SMS, four models exhibited statistically significant degradation (*p**≤ *0.025), with one of the largest drops observed for Proposed ST-GCN ($\Delta =-18.2\%$,* p*=0.003). Overall, phase-structured evaluation (Expert Selected) yields more consistent robustness under increased variability, while several higher-capacity temporal/spatio-temporal configurations are more sensitive to distribution shift.

**Table 7:** Accuracy robustness comparison between the original held-out test set (N=76) and the expanded merged test set (N=130).* p*-values are from a two-sided two-proportion Z-test on accuracy.

View	Model	Acc (76) (%)	Acc (130) (%)	*Δ * (%)	p-value	Status
Expert selected	Spatial GCN	86.9	85.5	− 1.4	0.772	Stable
	Spatial + LSTM	93.4	84.6	− 8.8	0.062	Stable
	AAGCN	99.0	87.0	− 12.0	0.004	Drop
	T-GCN	97.9	90.8	− 7.1	0.069	Stable
	Proposed ST-GCN	86.8	83.8	− 3.0	0.562	Stable
	Proposed T-GCN	88.7	85.7	− 3.0	0.575	Stable
SMS	Spatial GCN	83.2	76.4	− 6.8	0.255	Stable
	Spatial + LSTM	69.7	68.1	− 1.6	0.849	Stable
	AAGCN	92.9	82.6	− 10.3	0.025	Drop
	T-GCN	92.1	75.3	− 16.8	0.003	Drop
	Proposed ST-GCN	85.3	67.1	− 18.2	0.003	Drop
	Proposed T-GCN	89.5	75.5	− 14.0	0.014	Drop
All-frames	Spatial GCN	91.4	82.6	− 8.8	0.096	Stable
	Spatial + LSTM	66.8	57.2	− 9.6	0.149	Stable
	AAGCN	77.1	73.2	− 3.9	0.468	Stable
	T-GCN	87.4	67.8	− 19.6	0.002	Drop
	Proposed ST-GCN	84.7	64.1	− 20.6	0.002	Drop
	Proposed T-GCN	89.2	70.8	− 18.4	0.002	Drop

### Subject-independent evaluation

Table 8 reports the subject-independent leave-one-subject-out evaluation. In this stricter setting, each participant is held out entirely during testing. Held-out-subject accuracy ranged from about 50 to 74%, which is lower than the subject-dependent held-out video results. This indicates that the original video-level evaluation should be interpreted as subject-dependent performance rather than full cross-person generalization. Under SMS-RT selection, performance was comparable to or better than All-frames for several models, suggesting that sparse frame selection is not the main source of the cross-subject performance drop.

**Table 8:** Subject-independent leave-one-subject-out (LOSO) evaluation. Each held-out participant is tested after training on the remaining two participants. Accuracy is reported for both All-frames and SMS-RT inputs, with the SMS-RT selector retrained within each fold to avoid using held-out subject data during frame selection.

Model	Held-out S1		Held-out S2	
	All-frames	SMS-RT	All-frames	SMS-RT
Spatial GCN	70.0	69.8	65.8	55.7
Spatial + LSTM	50.4	64.5	56.2	56.2
AAGCN	58.7	64.5	53.1	51.8
T-GCN	52.9	69.4	56.2	56.2
Proposed ST-GCN	60.3	73.6	56.2	57.5
Proposed T-GCN	70.3	59.5	58.9	56.2

## Discussion

Deadlift form assessment poses unique challenges because minor deviations in hip or spine alignment can lead to major injury risks. Automated form assessment through video analysis offers a promising solution, yet accurately evaluating form from monocular RGB video remains a formidable challenge. The repetitive nature of deadlift movements produces redundant frames, inflating computational demands without contributing meaningful information. Our proposed framework, which integrates the SMS-RT frame selection method with graph convolutional networks, addresses these challenges by reducing redundancy, improving efficiency, and achieving competitive accuracy among the models evaluated in this study.

The SMS-RT method achieved an 89.8% frame reduction while preserving critical movement information. This efficiency stems from the inherent temporal redundancy in exercise videos and the hierarchical biomechanics of the deadlift. Unlike general action recognition, where every moment may carry unique information, deadlifts follow predictable phases—setup, eccentric lowering, concentric pull, and lockout—each with varying informational density. The setup and lockout phases are relatively static, while the concentric pull contains the dynamic, form‑critical moments. SMS-RT’s three-stage hierarchical process, powered by a ResNet50+TSN backbone, learns to identify these biomechanically significant moments and filter redundant frames. By targeting frames that minimize classification loss, SMS-RT consistently selects information-dense temporal windows where good and bad form diverge most clearly, achieving low variance in performance (mean loss 0.704 ± 0.050). This mirrors the intuition of expert trainers, who focus on key checkpoints like the initial pull, knee-level transition, and lockout, where errors such as hip hinge faults or spinal misalignment are most apparent. This targeted sampling aligns closely with expert evaluation strategies that prioritize the initial pull, knee-level transition, and lockout phases, explaining the minimal gap between SMS-RT and expert-selected frames (89.8% vs 92.0% F1-score).

A central component of the framework is the Adaptive Attention Graph Convolutional Network (AAGCN), which achieved the highest F1-score of 93.2% with SMS-RT. This performance may be attributed to its ability to learn task-specific joint connectivity patterns, overcoming the limitations of static skeleton-based models. Traditional graph methods rely on fixed anatomical priors, which may fail to capture the complex, dynamic relationships between body segments during a deadlift—for instance, the interplay between ankle positioning and spinal alignment or the coupling of shoulder blade stability and hip hinge mechanics. AAGCN’s adaptive topology learning discovers these non-obvious biomechanical dependencies, making it particularly effective for deadlift assessment, where subtle multi-joint coordination defines proper form. On the internal benchmark, AAGCN’s adaptive topology yielded the strongest F1-scores; however, its accuracy dropped significantly on the expanded external set (Table 7), indicating that this advantage did not fully transfer to broader capture conditions.

While AAGCN delivered the highest F1-scores given broader temporal coverage, the proposed ST-GCN and the proposed T-GCN achieved a different optimal point on the speed–accuracy frontier. Complementing SMS-RT, the proposed Spatio-Temporal Graph Convolutional Network (ST-GCN) and Temporal Graph Convolutional Network (T-GCN) models introduced a unified spatio-temporal graph architecture, achieving competitive performance with significantly reduced computational overhead. Unlike traditional approaches like Spatial+LSTM, which process spatial and temporal features sequentially, these models enable simultaneous message passing across spatial (within-frame joint relationships) and temporal (across-frame joint trajectories) edges. Because every node participates in dense spatio-temporal message passing within a compact window, curated six-frame inputs concentrate discriminative kinetics and reduce the dilution that can arise from long, redundant sequences. This design is ideally suited for deadlift assessment, where proper form hinges on both instantaneous joint configurations (e.g., hip–knee alignment) and their temporal evolution (e.g., consistent bar path). By eliminating separate processing stages, the proposed ST-GCN and the proposed T-GCN models avoid computational bottlenecks and enable joint optimization of spatial and temporal features, resulting in 0.4–0.5 s per-repetition model inference times. Their optimization for fixed-size temporal windows, such as those provided by SMS-RT or expert selections, ensures efficient feature extraction from strategically chosen frames, unlike AAGCN, which benefits from broader temporal coverage but requires more computational resources.

In contrast, the Spatial + LSTM model’s limitations highlight a fundamental architectural mismatch: recurrent sequence models flatten skeletal coordinates and thereby obscure anatomical connectivity. Capturing simultaneous multi-joint relationships (e.g., hip–knee–ankle chain, shoulder–spine–pelvis alignment) is essential for deadlift assessment; relying on temporal modeling alone yielded a lower F1-score (74.5% with SMS-RT), indicating the advantage of graph-based approaches for capturing complex deadlift kinematics.

The consistent advantage of SMS-RT over random frame sampling further supports its design. Random sampling treats all temporal moments as equally informative, ignoring the biomechanical structure of deadlifts, resulting in high variance and inconsistent performance. In contrast, SMS-RT’s learned understanding of temporal structure ensures it consistently targets critical phases, providing models with high-quality input data. This noise reduction capability explains why SMS-RT sometimes outperformed full-frame processing, particularly for AAGCN, where F1-score improved from 81.1 to 93.2% . By filtering out redundant frames from setup or completion phases, SMS-RT prevents overfitting on uninformative patterns, allowing models to focus on discriminative biomechanical features. Empirically, SMS-RT also reduced cross-fold variability relative to all-frames and random baselines, indicating more stable generalization under constrained input budgets.

The framework has practical implications for efficient video-based exercise assessment. By reducing computational demands by 89.9%, SMS-RT enables real-time processing on standard hardware, reducing the computational cost of processing long video sequences that plagues traditional video-based assessment. The graph-classification stage operates on a fixed-size six-frame input and is therefore near constant-time; the upstream frame-selection pass scales with clip length, so end-to-end latency grows modestly with video duration (Table 5). The sub-second model inference times (0.4–0.5 s; approximately 0.8 s end-to-end) open the door to immediate form guidance, broadening access to automated coaching for lifters without expert supervision. Moreover, The framework’s modular design also suggests extensibility to other resistance exercises, supporting scalable, automated fitness monitoring tools. The latency results were measured on a desktop GPU rather than on a mobile or edge device; therefore, smartphone or embedded deployment feasibility remains future work.

Despite these advances, limitations should be considered. The study’s reliance on a narrow demographic—three male participants aged 19–25—limits generalizability, as deadlift biomechanics vary across body types and experience levels. This limited cohort also includes little variation in sex and body proportions, so broader generalization across populations remains a limitation. For instance, individuals with longer torsos may exhibit different hip and spine angles during proper form. Form quality labels for the internal dataset were assigned by a single certified trainer; therefore, labeling bias due to subjective judgment remains a limitation of this study. Although an expanded evaluation set was constructed by merging the held-out internal test split with an external in-the-wild set reviewed using three-annotator consensus, the statistically significant degradation observed for several SMS and All-frames configurations in Table 7 indicates a meaningful distribution shift under broader capture conditions. In contrast, the relative stability of the Expert Selected view suggests that phase-defining kinematic checkpoints may provide a more robust basis for assessment under increased variability. These findings show that the method is not yet fully robust to unconstrained real-world variation, and that larger, more diverse training data are still needed before broad deployment claims can be made. The subject-independent LOSO evaluation further supports this cautious interpretation: when a participant is held out entirely, accuracy decreases relative to the video-level split, confirming that the original results should be interpreted as subject-dependent performance rather than full cross-person generalization. However, SMS-RT remains comparable to or better than All-frames for several models, suggesting that sparse frame selection itself is not the main cause of the cross-subject performance drop. Moreover, deadlift technique errors often exist along a continuous risk spectrum, whereas the present task is formulated as a binary good/bad classification, limiting fine-grained coaching and clinical interpretation. Additionally, the use of 2D pose estimation introduces systematic errors, as depth information critical for assessing bar path or spinal curvature in the sagittal plane is absent in monocular video. The proposed framework prioritizes scalable real-time deployment on commodity RGB cameras; while 3D motion capture or depth sensors can improve geometric accuracy, they reduce practicality for typical gym settings. Accordingly, the system captures hip–spine alignment errors only as they appear in 2D projections and cannot recover true 3D spinal curvature or a metrically accurate bar path. Pose uncertainty caused by occlusion, non-sagittal camera placements, and clothing-induced keypoint errors may compound misclassification risk; robustness to these factors remains a priority for future deployments. Future work should explore multi-view capture or sensor fusion to enhance accuracy and robustness, alongside expanding datasets to include diverse populations and real-world conditions like variable camera angles or background clutter and multi-annotator labeling with inter-rater reliability analysis.

## Conclusion

This work presented an efficient, vision-based framework for deadlift form assessment that jointly optimizes informational efficiency and classification performance. By integrating the SMS-RT frame selection pipeline with graph-based models, the system reduced input redundancy by 89.8% while preserving movement-critical information and delivering high accuracy with 0.4–0.5 s per-repetition model inference (approximately 0.8 s end-to-end), enabling practical deployment on commodity hardware. Overall, the results demonstrate that selecting a small set of information-rich frames and modeling pose dynamics with graph-based networks enables accurate and efficient deadlift form assessment without depth sensors or wearables. Because the system relies on monocular 2D pose, it should be interpreted as 2D projection-based form screening rather than direct 3D biomechanical measurement.

Limitations include sensitivity to pose-estimation noise and viewpoint/occlusion effects, and the current dataset focuses on a single exercise and labeling protocol. The internal dataset is a single-site pilot of 377 clips from three young male participants, which limits generalization across sex, body morphology, and training experience. Latency was measured on a desktop GPU rather than a mobile or edge device, so on-device deployment remains future work. Future work will expand data diversity (camera angles, populations, gym conditions), improve robustness under occlusions/lighting variation, and extend the framework to multi-class error diagnosis and additional resistance exercises.

## Data Availability

The datasets generated during and/or analyzed during the current study are available from the corresponding author on reasonable request.

## Code availability

The source code for the SMS-RT frame-selection pipeline, the graph-construction module, and all graph neural network models evaluated in this study is openly available on Zenodo (DOI: https://doi.org/10.5281/zenodo.20802956). The deposited snapshot contains the code and configuration files required to reproduce the reported experiments.
